# Uncharted Source of Medicinal Products: The Case of the *Hedychium* Genus

**DOI:** 10.3390/medicines7050023

**Published:** 2020-04-28

**Authors:** Wilson R. Tavares, Maria do Carmo Barreto, Ana M. L. Seca

**Affiliations:** 1 cE3c—Centre for Ecology, Evolution and Environmental Changes/Azorean Biodiversity Group & Faculty of Sciences and Technology, University of Azores, Rua Mãe de Deus, 9501-321 Ponta Delgada, Portugal; 2 LAQV-REQUIMTE, Department of Chemistry, University of Aveiro, 3810-193 Aveiro, Portugal

**Keywords:** *Hedychium*, traditional medicine, coronarin D, villosin, anti-acetylcholinesterase, antidiabetic, anti-inflammatory, antimicrobial, antioxidant, antitumor

## Abstract

A current research topic of great interest is the study of the therapeutic properties of plants and of their bioactive secondary metabolites. Plants have been used to treat all types of health problems from allergies to cancer, in addition to their use in the perfumery industry and as food. *Hedychium* species are among those plants used in folk medicine in several countries and several works have been reported to verify if and how effectively these plants exert the effects reported in folk medicine, studying their essential oils, extracts and pure secondary metabolites. *Hedychium coronarium* and *Hedychium spicatum* are the most studied species. Interesting compounds have been identified like coronarin D, which possesses antibacterial, antifungal and antitumor activities, as well as isocoronarin D, linalool and villosin that exhibit better cytotoxicity towards tumor cell lines than the reference compounds used, with villosin not affecting the non-tumor cell line. Linalool and α-pinene are the most active compounds found in *Hedychium* essential oils, while β-pinene is identified as the most widespread compound, being reported in 12 different *Hedychium* species. Since only some *Hedychium* species have been investigated, this review hopes to shed some light on the uncharted territory that is the *Hedychium* genus.

## 1. Introduction

Since the beginning of the history of mankind there was always a connection between plants and human health, as they were used as food and medicines [1]. The traditional herbal medicine outlined the foundations from which modern medicine developed and is still largely practiced around the world [2], particularly in Asian and developing countries [3,4]. This popular knowledge, also known as folk medicine, gives a good indication to scientists looking for sources of new compounds with pharmaceutical potential. Thus, medicinal plants and their derived natural compounds have become an increasing topic of investigation and interest [5,6].

According to “The Plant List” database [7], the genus *Hedychium* (Zingiberaceae family) comprises 93 species with accepted scientific plant names that, with the exception of *Hedychium peregrinum* N.E.Br. that is endemic to Madagascar [8], are native to wooded habitats in tropical and temperate Asia (i.e., China, Indian subcontinent and Southeast Asia) [8,9,10]. Members of this genus are well distributed worldwide, being easily found particularly throughout tropical Asia, Australia, Fiji, New Caledonia, New Guinea, New Hebrides, Samoa and the Solomon Islands [8,10,11], with some species being considered invasive in some places: e.g., *Hedychium coronarium* J. Koenig in Brazil [12] and *Hedychium gardnerianum* Sheppard ex Ker-Gawl. in Azores Archipelago [13] and Hawaii [14].

*Hedychium* species are medium-size rhizomatous perennial monocotyledonous plants that can be easily recognized by their characteristic striking foliage and terminal spikes that produce diversified numerous short-lived flamboyant flowers with several hues and fragrances varying depending on the species [15]. These features give them a high ornamental value, being cultivated worldwide mostly for this purpose and for its use in the perfumery industry, since, besides the aromatic flowers, *Hedychium* species rhizomes also originate strongly scented oils [16,17].

The use of *Hedychium* species in folk medicine is common in several countries since they are easily harvested directly from nature or obtained at local markets [18]. These plants are reported to possess analgesic, antimicrobial, antidiabetic, anti-inflammatory, antitumor, anti-allergic, anthelmintic and antioxidant properties [19,20,21,22]. In Table 1, it is summarized the different *Hedychium* species with reported traditional medicinal use in literature over different geographic areas.

In addition to the traditional medicinal uses stated in Table 1, *Hedychium* species are also included in the diet of some populations, like in Thailand where the flowers of *Hedychium forrestii* Diels can be boiled to become a beverage [45] or in India where the fruit of *H. spicatum* may be cooked and eaten with lentils in savory dishes [42]. Moreover, the rhizome of *H. coronarium* is also included in the diet of some populations of South East Asia, being consumed as a vegetable or as a food flavoring spice [46].

The traditional uses mentioned above show that several *Hedychium* species are used to treat a wide spectrum of diseases. These uses also show that *Hedychium* species should be considered as promising sources of new bioactive natural compounds and that is why these species have been the target of research by the scientific community. In recent years, several studies have been published on the phytochemical characterization of *Hedychium* species, as well as on the evaluation of the biological activities exhibited by their organic extracts, essential oils and pure compounds, with some of them showing very interesting results. Recently, literature reviews have been published focusing only on specific species, i.e., *H. coronarium* [20,47] and *H. spicatum* [21,48]. This work aims to update the available information that were not mentioned in the previous reviews, as well as involving all the other *Hedychium* species, their bioactivities and their bioactive isolated compounds. The research for this review was made combining the terms *Hedychium*, phytochemical and biological activities in the databases Web of Science, PubMed and Scopus and were considered only the published works involving *Hedychium* species whose binominal Latin name is an accepted name on the The Plant List database [7].

## 2. In Vitro and In Vivo Activities of *Hedychium* Extracts and Essential Oils

Taking into account the traditional uses of *Hedychium* species, several works have been carried out to elucidate how effectively plants can exert the reported biological effects. The following is a compilation and discussion of the most current works on this subject, in which essential oils and extracts of *Hedychium* species are studied and their biological activities are ascertained.

### 2.1. Anti-Acetylcholinesterase

The inhibition of the enzyme acetylcholinesterase (AChE) is one of the pathways to countering the cholinergic deficit associated with cognitive dysfunction diseases like in Alzheimer’s disease [49]. Arruda and colleagues [50] showed that the leaf essential oil of *H. gardnerianum* collected from four different locations could inhibit AChE action, mainly mixed inhibition, presenting IC_50_ values ranging from 1.03 ± 0.14 mg/mL to 1.37 ± 0.27 mg/mL, a value not statistically different from the value displayed by the AChE inhibitor standard compound α-pinene that presented an IC_50_ value of 1.43 ± 0.07 mg/mL. This work showed no statistically significant difference between the activity of samples taken in different geographical areas [50].

### 2.2. Antidiabetic

Deficiency in insulin secretion, insulin action or both, results in chronic hyperglycemia, the main characteristic of diabetes mellitus [51], the main treatment to this condition being the use of anti-diabetic drugs that can control glucose levels in the blood [52].

An in vivo study [53] was carried out to assess the effect of *H. coronarium* aqueous extract to lower blood glucose level in induced-type 2 diabetes mellitus (T2DM) animal models (streptozotocin (STZ)-induced T2DM Wistar rats and C57BKS^db/db^ mice, a mice model with a mutation that results in chronic hyperglycemia, pancreatic beta cell atrophy, low insulin level and obesity). After 28 days, the daily dose of *H. coronarium* aqueous extract (8.928 mg/kg for the STZ-induced T2DM rats and 17.71 mg/kg for the C57BKS^db/db^ mice) significantly increased glucose tolerance in both diabetic models, when compared with the group treated with distilled water (control group). In addition, the treatment also helped to maintain optimal β-cell structure, moderately increased insulin, improved the lipid profile and decreased aldosterone level in STZ-induced T2DM model.

In another in vivo assay [54], after 14 days of treatment, using an oral dose of 0.3 mL of essential oil from rhizomes of *H. spicatum*, was observed the reduction of blood glucose and urea levels in rats with diabetes induced by intraperitoneal injection of a solution of alloxan monohydrate (150 mg/kg). This result is similar to those obtained in the group of rats treated with the reference drug glibenclemide. Furthermore, it was noticed that the Islets of Langerhans regained their normal shape after the treatment period [54].

### 2.3. Anti-Inflammatory

Inflammation is a vital defense mechanism that works to ensure good health [55], but uncontrolled inflammation may lead to serious repercussions [56] and so it is important to continue research into products that can help in its control.

An in vivo study [57] with rats demonstrated the anti-inflammatory effect of a single oral dose (200 mg/kg) of aqueous and ethanolic extracts of *H. spicatum* rhizome against carrageenan-induced paw edema. Measurements of the edema volume were taken in a successive interval of 1 h, 2 h and 3 h and significant decrease in paw edema volume was detected since the beginning, with the aqueous extract reporting a 28.10% decrease in inflammation and the ethanolic extract a 25.62% decrease in inflammation. Although none of the extracts performed as well as the positive control compound indomethacin (41.32% decrease in inflammation), they both proved to present no acute toxicity in a concentration as high as 2000 mg/kg, with the rats never showing secondary toxic effects like coma, convulsion, salivation, increased motor activity or death. This dose of 2000 mg/kg was previously utilized in a similar work [58] where the ethanolic extract of *H. spicatum* reported a 55.54% of anti-inflammatory activity inhibition against carageenan-induced edema in rats.

### 2.4. Antimicrobial

A healthy human body is a symbiosis between human and microbial components [59]. However, sometimes that symbiotic balance can be disturbed, and human health can be impaired by pathogenic microorganisms (i.e., bacteria, fungi, parasites or viruses), the use of effective antimicrobial drugs being needed to restore health normality [60,61].

Noriega et al. [62] showed that, among five different plants, the essential oil of *H. coronarium* rhizome exhibited the most relevant antibacterial activity against *Listeria grayi* (MIC value = 0.45 mg/mL) and *Streptococcus mutans* (MIC value = 0.18 mg/mL) and even against the Gram-negative bacteria *Klebsiell oxytoca* (MIC value = 0.90 mg/mL). The authors point out the compounds 1,8-cineole and terpinen-4-ol as responsible for the reported activity [62]. In another work [63], *H. coronarium* leaves essential oil was also pointed out to have antibacterial activity against different bacterial strains, i.e., *Escherichia coli* (MIC value = 3.90 μL/mL), *Staphylococcus aureus* (MIC value = 7.81 μL/mL) and *Pseudomonas aeruginosa* (MIC value = 15.62 μL/mL). These two works are presented here also as examples of two constraints which are common in a variety of scientific papers. First, no work reports, as a comparative term, the activity exhibited by a standard antibacterial compound, determined under the same experimental conditions as the essential oil samples. Without these data it is very difficult to assess the true potential of the samples tested. Second, the MIC values are expressed in non-comparable units. Fortunately, one of the works [62] presents the density of the essential oil, making it possible to convert one of the sets of results [against *Listeria grayi* (MIC value = 0.50 μL/mL), *Streptococcus mutans* (MIC value = 0.20 μL/mL), *Klebsiell oxytoca* (MIC value = 1.0 μL /mL)], allowing to conclude that the essential oil from rhizome is more active as antibacterial agent than leaves essential oil. Regrettably, some papers do not present enough experimental data to allow a unit conversion. Additionally, Ray et al. [64] reported that the essential oil extracted from the rhizome of *H. coronarium* is an effective antifungal agent since it exhibited activity against *Candida albicans* (MIC = 3.12 μg/mL), *Aspergillus flavus* and *Fusarium oxysporum* (MIC value of 6.25 μg/mL for both species), these MIC values being much lower than those reported for antibacterial activity by Noriega et al. [62].

Another work [65] found that 20 μL of *Hedychium matthewii* S. Thomas, B. Mani & S. J. Britto rhizome essential oil could be as effective as 30 μg of the standard antibiotic amoxicillin, since it exerted nearly the same growth inhibition effect against several strains of Gram- positive and Gram-negative bacteria (*viz. Bacillus cereus*, *Staphylococcus aureus*, *Enterobacter aerogens*, *Salmonella paratyphi*, *Salmonella typhii*, *Escherichia coli*, *Vibrio parahaemolyticus*, *Proteus vulgaris*, *Klebsiella pneumoniae* and *Pseudomonas aeruginosa*). Furthermore, it could be pointed out that *Streptococcus haemolyticus* and *Vibrio cholerae* were more susceptible towards the essential oil (20 μL) than towards amoxicillin (30 μg).

The activity of *H. spicatum* flowers essential oil was evaluated against the Gram-negative bacteria *Borrelia burgdorferi* in stationary phase cycle and it was found out that a 0.1% (v/v) essential oil concentration could eradicate *B. burgdorferi* (100 μL) with no regrowth [66]. This is one of the few published works that evaluates the antibacterial activity in the stationary-phase of growth.

A different work [67] found that a combination treatment using essential oil of *H. spicatum* rhizomes and *γ*-radiation was effective against *Fusarium graminearum*, inhibiting both the fungal growth in maize grains and the production of the toxic mycotoxins deoxynivalenol and zearalenone in a dose-dependent way, with a complete inhibition at the concentration of essential oil 1.89 mg/g and 4.1 kGy of *γ*-radiation. Combinational treatment proved to be better than individual treatment, since complete inhibition of *F. graminearum* required the essential oil concentration of 3.15 mg/g or 6 kGy of *γ*-radiation.

It is not just the essential oils of *Hedychium* species that have been evaluated concerning antimicrobial activity. Arora and Mazumder [68] evaluated the activity of *H. spicatum* rhizomes methanolic extract and the antibiotic ciprofloxacin against different bacterial strains (*viz. Shigella boydii*, *Shigella soneii*, *Shigella flexneri, B. cereus, V. cholerae*, *E. coli, S. aureus, Ps. aeruginosa* and *K. pneumoniae*) at the concentrations of 200 to 1200 μg/mL. The results showed a similar inhibition effect for both antibiotic and extract, *B. subtilis* being the bacteria with greater susceptibility to the extract and antibiotic.

Another work [69] evaluated the anthelmintic activity of methanolic, ethanolic, hydromethanolic, hydroethanolic and aqueous rhizome extracts of *H. spicatum* against *Hemonchus contortus*, with the results showing that the methanolic extract were as effective as the positive control compound thiabendazole on time taken for paralysis and time taken for death (tested concentrations 20, 40 and 60 mg/mL).

### 2.5. Antioxidant

Oxygen metabolism is fundamental for human life but its reaction products, like reactive oxygen species (ROS), can increase oxidative stress, causing damage to cells and tissues [70] that, with time, leads to the development or aggravation of several chronic diseases [71]. Thus, therapeutic antioxidant agents are key to mitigate the oxidative stress impact in human health, with natural plant-derived products being the main investigation focus of search [72].

Noriega et al. [62] evaluated the antioxidant activity of the essential oil extracted from the rhizome of *H. coronarium*, reporting IC_50_ values of 9.04 ± 0.55 mg/mL and 2.87 ± 0.17 mg/mL for 1,1-Diphenyl-2-picrylhydrazyl (DPPH) and 2,2′-azino-bis(3-ethylbenzothiazoline-6-sulphonic acid (ABTS) assays, respectively. In a similar work, Ray and colleagues [64] also evaluated the antioxidant activity of the essential oil from *H. coronarium* rhizome, but from ten distinct regions of India, obtaining activity values higher than those indicated in the work of Noriega et al. [62] (IC_50_ values range from 0.57 to 2.19 mg/mL for the DPPH assay; and 0.12 to 0.67 mg/mL for the ABTS assay), but lower than the positive control 2,6-di-tert-butyl-4-methylphenol (BHT) (IC_50_ = 0.12 ± 0.01 mg/mL on the DPPH assay, and 0.08 ± 0.01 mg/mL on the ABTS assay). It could be pointed out that Ray et al. [64] also demonstrate, very clearly, that the geographical origin of the samples is a relevant variable for the level of activity displayed. The same conclusion can be drawn from the results obtained by Arruda et al. [50], where the DPPH antioxidant activity of *H. gardnerianum* leaf essential oil collected from four different locations ranged from EC_50_ = 8.46 ± 0.90 μg/mL to 31.14 ± 2.70 μg/mL (EC_50_ = 31.00 ± 0.19 μg/mL for BHT). In a more recent work, Ray et al., [73] studied the antioxidant activity of *Hedychium greenii* W. W. Smith. and *Hedychium gracile* Roxb. rhizomes essential oils by the same methodology (DPPH and ABTS assays), with *H. greenii* showing higher antioxidant activity (IC_50_ values of 16.73 ± 0.19 μg/mL for DPPH and 12.18 ± 0.16 μg/mL for ABTS assays) than *H. gracile* sample (IC_50_ values of 46.94 ± 0.6 μg/mL for DPPH and 31.13 ± 0.29 μg/mL for ABTS assays), and slightly higher than the positive control BHT (IC_50_ = 18.94 ± 0.3 μg/mL and IC_50_ = 14.21 ± 0.27 μg/mL for DPPH and ABTS assays, respectively). These results [73], when compared with those obtained in the works mentioned above [50,64], show that the level of antioxidant activity of essential oils exhibits variability between different *Hedychium* species (IC_50_ values range from 8.46 to 2190 μg/mL) higher than geographical variability (IC_50_ values range from 0.57 to 2.19 mg/mL for the DPPH assay).

Zhao et al. [74] compared essential oils and ethanolic extracts from rhizomes of different species from the Zingiberaceae family in terms of its antioxidant capacity by DPPH assay. The ethanol extracts of *H. coronarium* and *H. gardnerianum* proved to be the best antioxidant samples presenting IC_50_ values of 0.94 μg/mL and 1.59 μg/mL, respectively, even better than the reference compounds trolox (IC_50_ = 10.19 μg/mL) or ascorbic acid (IC_50_ = 8.37 μg/mL). Essential oils of these plants were also tested but unfortunately the authors presented the results as a graphic which does not allow the reading of numerical values of antioxidant activity.

Usha et al. [75] compared the hydromethanolic rhizome extract of different species also from Zingiberaceae family in terms of its antioxidant capacity and found out that *Hedychium* sp. reported the best results, with the lowest IC_50_ value on DPPH assay (36.4 μg/mL). This activity was correlated with its high phenol and flavonoid content. Unfortunately, the authors do not specify neither the *Hedychium* species that was used nor the IC_50_ value of the ascorbic acid used as positive control, which makes impossible to compare with other published works.

Another work [69] evaluated, through ABTS, DPPH and nitric oxide (NO) free radical scavenging assays, the antioxidant activity of methanolic, ethanolic, hydromethanolic, hydroethanolic and aqueous rhizome extracts of *H. spicatum*. The results showed the methanolic extract as the most antioxidant extract, presenting the lowest EC_50_ values for all the assays (EC_50_ ABTS value = 24.93 mg/mL, EC_50_ DPPH value = 8.31 mg/mL and EC_50_ NO value = 3.57 mg/mL). However, this extract is much less active than the positive control ascorbic acid (EC_50_ = 1.63 mg/mL to ABTS assay, EC_50_ = 0.049 mg/mL to DPPH assay and EC_50_ = 0.10 mg/mL to NO assay) and since the extract EC_50_ values are very high, it should be considered an inactive extract.

In an in vivo study, Choudhary and Singh [76] demonstrated the antioxidant potential of *H. spicatum* rhizome, since an improvement in the oxidative stress state of white leghorn cockerels (*Gallus gallus domesticus*) was observed after the rhizome powder was added to the animal diet, following chronic exposure to indoxacarb.

### 2.6. Antitumor

Cancer is a complex disease that is a major cause of death worldwide [77], with several treatments but no cure [78]. In the light of the aggressive and not always effective treatments in current medicine, the demand for safer and better anticancer compounds have turned the search to natural products as another therapeutic approach to cancer [79].

Ray and colleagues [80] demonstrated the antiproliferative time-dependent effect of *H. coronarium* rhizome ethanol extract against human cervical carcinoma HeLa cells, without affecting the viability of non-tumor human umbilical vein endothelial cells (HUVEC). After 24, 48 and 72 h of incubation, the observed IC_50_ values were 17.18 ± 0.46, 15.32 ± 0.68 and 12.57 ± 0.32 μg/mL, respectively. Although the positive control drug camphothecin presented a far greater inhibitory effect against HeLa cells (IC_50_ values of 0.82 to 0.98 μg/mL), it is also more toxic to the HUVEC cells (IC_50_ value for 24 h = 10.13 ± 0.62 μg/mL) than the *H. coronarium* ethanol extract (IC_50_ value for 24 h > 320 μg/mL), which means that the extract presents a higher selective cytotoxicity. In addition, the same study shed some light on the mechanism whereby the extract exerts its antitumor activity. It denotes the modulation of the expression of proapoptotic and antiapoptotic protein levels together with an increase of ROS generation and consequent oxidative stress induction in HeLa cells that led to an apoptosis-mediated G1 phase cell arrest as the main cause of HeLa cells migratory capacity inhibition.

In another study [81], the methanolic extract of *H. spicatum* rhizomes was described as possessing a dose-dependent cytotoxicity activity against human liver hepatocellular carcinoma cell line HepG2, testing concentrations in the range of 25 to 3000 μg/mL. The concentrations tested and the IC_50_ value (281.917 μg/mL) are very high, and the authors do not provide the cytotoxicity of a positive control nor do they evaluate the effects of such concentrations on non-tumor cells. The results obtained in the studies performed in these conditions, should be considered with many reservations as the effects observed using such high concentrations are non-specific. On the other hand, the researchers should take into account that 20 µg/mL is the limit established by the National Cancer Institute to consider an extract active enough to justify continuing its study [82], so the tested extract should be considered inactive against HepG2 cells line.

The in vitro cytotoxicity, by 3-(4,5-dimethylthiazol-2-yl)-2,5-diphenyltetrazolium bromide (MTT) assay, of *H. spicatum* rhizome chloroform extract was assessed against colorectal adenocarcinoma (Colo-205) cell line, human epidermoid carcinoma (A-431) cell line, human breast adenocarcinoma (MCF-7) cell line, human lung adenocarcinoma (A549) and Chinese hamster ovary (CHO) cell lines [83]. The results show that the extract presented cytotoxicity against all cell lines exhibiting IC_50_ values ranging from 37.45 ± 0.90 µg/mL to 63.21 ± 1.19 µg/mL, including against non-tumor cell line CHO (39.52 ± 0.06 µg/mL), indicating that the *H. spicatum* rhizome chloroform extract have small potential as a good anticancer drug since it affected in a similar way both tumor and non-tumor cell lines. Results like these shows how difficult it is to find an ideal anti-tumor drug that affect only the tumor cells, leaving the non-tumor cells undamaged. In addition, it would have been interesting if the authors had also tested a reference compound, since it would have enriched their work.

### 2.7. Hepatoprotective

The liver is a vital organ, capable of detoxifying the body from endogenous and/or exogenous substances detrimental to the organism, and which is responsible for the regulation of diverse functions and physiological processes, such as the metabolism of carbohydrates and fats and the secretion of bile [84]. Exposure to drugs and chemicals can cause liver injury which, taking into account all the functions inherent to the liver, is a major health problem [85]. Thus, compounds that can protect the liver, stimulate hepatic function or help to regenerate hepatic cells, while simultaneously being less toxic and more effective are of great interest, with natural sources being identified as good search option [86].

A study [87] indicated that *H. spicatum* possess hepatoprotective properties since its three rhizome extracts (methanolic, ethanolic and aqueous) exerted protection on HepG2 cells against paracetamol-induced toxicity. The IC_50_ values were 282, 356 and 515 μg/mL for the methanolic, ethanolic and aqueous extracts, respectively, which translates in a cytoprotection percentage of 16%, 13% and 9%, respectively. Compared to the 19% cytoprotection provided by the control substance silymarin (IC_50_ = 110 μg/mL), the hepatoprotective effect of the extracts is not huge but it is worth mentioning at least the methanolic extract.

A study which was also carried out to evaluate the potential hepatoprotective effect was the in vivo study [88]. where cockerels were fed for 16 weeks with rhizome powder of *H. spicatum*, while simultaneously receiving a dose of indoxacarb intended to cause chronic toxicity. The results of the liver analysis show that, when compared with the control group (indoxacarb administration without the added *H. spicatum* rhizome powder to the cockerels diet), *H. spicatum* rhizome ameliorated the damages caused in cockerels by indoxacarb in the duration of the experiment. Apparently, the treatment with *H. spicatum* modulated the expression levels of several different hepatic genes, such as those involved in metabolization of indoxacarb (cytochrome P450 1A1), in the immune system (interleukin 6 (IL-6)) and in antioxidant function (catalase (CAT), superoxide dismutase (SOD) and glutathione peroxidase (GPx)).

### 2.8. Insecticide

Control of mosquito population is crucial, particularly in developing countries, since they act as vectors of several pathogens and parasites responsible for various worrisome diseases, e.g., dengue, filariasis, malaria, West Nile or yellow fever [89,90]. In order to reduce or eliminate the human contact with the vector, a wide range of methods exists with insecticides being a top choice in case of mosquitoes [91]. However, with insecticide resistance being a problem in recent years [92], the search for better substances with insecticide potential is imperative.

Kalimuthu and colleagues [93] carried out an interesting work where *H. coronarium*-synthesized silver nanoparticles (AgNPs) were produced and their toxicity towards larvae and pupae of the dengue vector *Aedes aegypti* was assessed, as well as their synergy with *Mesocyclops formosanus* predation over *A. aegypti* larvae. The toxicity of aqueous *H. coronarium* rhizome extract was also assessed. The results indicate that both *H. coronarium* formulations tested, aqueous rhizome extract and AgNPs, were toxic against *A. aegypti* in a dose-dependent manner. Aqueous *H. coronarium* rhizome extract caused toxicity with LC_50_ values from 0.688% against larval instar I to 1.882% dose against pupae stage of *A. aegypti*, while AgNPs demonstrated its toxicity with LC_50_ values varying from 24.264 ppm for larval instar I till 348.68 ppm for pupae of *A. aegypti*. Once again, we are faced with a work whose authors express results in non-comparable units and do not provide the necessary data for their conversion, significantly reducing the impact of this work. Nevertheless, AgNPs were found to be stable over time in aquatic environment and since a positive synergy was reported with *M. formosanus* predation on young *A. aegypti* larvae, its combined use could lead to a higher efficacy in removing the larval population of dengue mosquitoes from aquatic areas.

In another work [94], *Hedychium larsenii* M. Dan and C. Sathish Kumar rhizomes essential oil was evaluated regarding its toxicity against larvae of mosquito vectors of diseases, namely *Anopheles stephensi* (malaria), *A. aegypti* (dengue) and *Culex quinquefasciatus* (St. Louis encephalitis). The results demonstrate that the essential oil exerted larvicidal activity over the different larvae with the LC_50_ values of 82.02, 88.60 and 96.40 μg/mL for *A. stephensi*, *A. aegypti* and *C. quinquefasciatus*, respectively. Again, the lack of a tested reference compound impairs any conclusion taken from these results.

## 3. Secondary Metabolites from *Hedychium* Species and Its Activities

**Figure 1:**
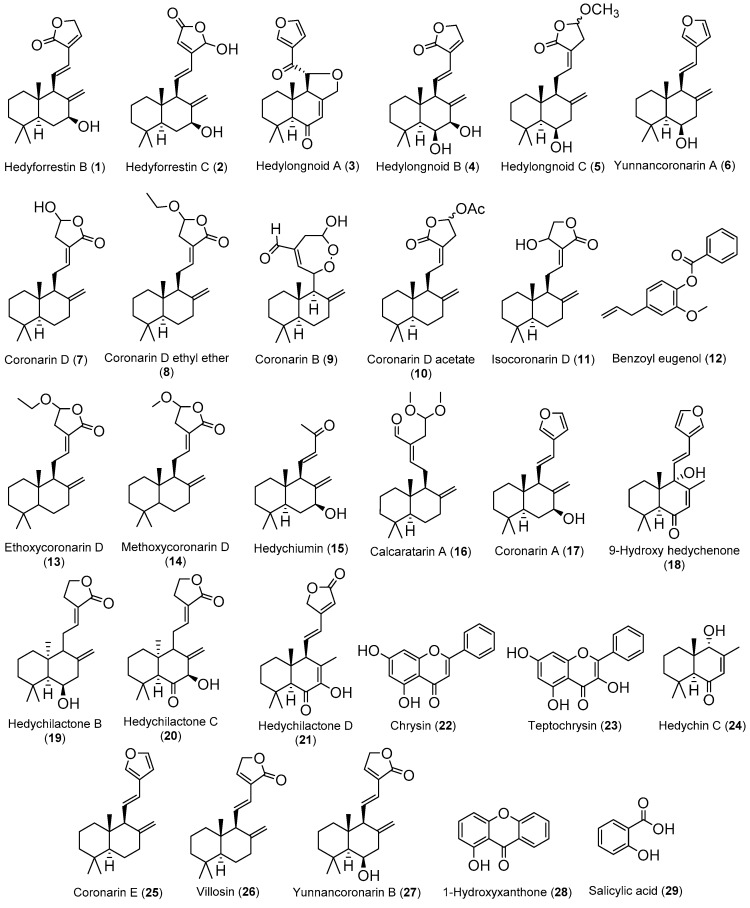
Chemical structure of the compounds referred on Table 2.

The diverse bioactivities observed on different *Hedychium* species/extracts are intrinsically linked to the compounds present in each one, so the need and interest in the phytochemical study of these extracts/species becomes clear. Several relevant works managed to isolate compounds from *Hedychium* extracts and carried out different assays to ascertain the bioactive potentials of those compounds. In Table 2 the compounds isolated from *Hedychium* extracts are gathered, as well as their bioactivities and the *Hedychium* species where they have already been identified. A figure with the chemical structures of the compounds (Figure 1) listed in this table is present after Table 2. It should be clarified that, for each compound in Table 2, only the highest activity value for each activity from each reference is presented, with some values converted from µg/mL to µM to facilitate comprehension and comparison of the different activities.

Taking the information of Table 2 into account, it is possible to identify that *H. coronarium* provided the highest number of isolated compounds and that the antitumor activity is the most reported bioactivity in the above-mentioned studies. On the other hand, the labdane-type diterpene is the most frequent family of compounds in the genus *Hedychium*, and some flavonoids and simple phenolic compounds are also identified.

Villosin (**26**) can be pointed out as the most promising antitumor compound, since it presented a highest and selective cytotoxicity against NCI-H187 cell line with an IC_50_ value of 0.40 µM, without toxicity against the non-tumor Vero cell line at 166.42 μM and presenting better results than the positive control compound ellipticine (i.e., IC_50_ value against NCI-H187 of 1.79 µM and IC_50_ value against Vero of 7.47 µM). Coronarin D (**7**) appears also as one interesting compound, since recent works report its antibacterial activity against *B. cereus* to be better than the positive control oxacillin.

In addition to these compounds, hedyforrestin B (**1**) and hedyforrestin C (**2**) should also be noted, since their antitumor activities against the NCI-H187 cell line are slightly lower (less than 1.7 times) than that shown by the reference compound ellipticine and with selectivity indices of 14.5 and 4.8, respectively.

On the other hand, compound isocoronarin D (**11**) should be highlighted since it exhibits activity against a broad spectrum of tumor cell lines (i.e., A549, human cervical carcinoma (HeLa), human hepatocellular carcinoma (HepG2), human acute promyelocytic leukemia (HL-60), human cholangiocarcinoma (HuCCA-1), human epidermoid carcinoma (KB), human breast adenocarcinoma (MDA-MB-231), human acute lymphoblastic leukemia T-lymphoblasts (MOLT-3), mouse lymphoma neoplasm (P388), human hepatocellular carcinoma (S102) and human hormone-dependent breast cancer (T-47D)), with IC_50_ values between 2.14 to 36.1 µM, better than etoposide or doxorubicin which are toxic only to some of these cell lines, and being more active against HepG2 (IC_50_ = 16.6 µM) than the reference compound etoposide (IC_50_ = 23.8 µM) [98].

Bearing in mind that all these compounds have hydroxyl groups and double bonds in their chemical structure, it is suggested that these compounds could be lead compounds, and researchers in the field of medicinal chemistry should use these labile functional groups to carry out structural modifications, in order to obtain more active derivatives and to determine the structure/activity relationships.

In addition, there are some works which require a critical analysis. Zhao and colleagues [96] isolated six labdanes from *H. longipetalum* rhizome that exhibited NO production inhibitory effects in lipopolysaccharides (LPS) and interferon gamma (IFN-γ)-induced murine macrophages RAW 264.7 cell line. The most active compound is yunnancoronarin A (**6**) (IC_50_ = 1.86 µM), but less active than the positive control carbobenzoxy-Leu-Leu-leucinal (MG132) (IC_50_ = 0.17 µM). Unfortunately, the authors do not mention the extraction and chromatographic procedures they carried out to isolate these compounds, which would have been a valuable information.

Another study that looks promising, but which actually shows very questionable results, is the one carried out by Kiem and colleagues [37]. They isolated compounds from rhizomes of *H. coronarium* methanol extract and investigated their anti-inflammatory potential through inhibition of pro-inflammatory cytokines production in LPS-stimulated bone marrow-derived dendritic cells (BMDC). The results are not acceptable and do not allow to infer conclusions since they are presented with associated standard errors greater than 20% (e.g., IC_50_ IL-6 inhibition value = 7.57 ± 2.02 µM) and in some cases close to 100% (e.g., IC_50_ IL-12p40 inhibition value = 0.19 ± 0.11 µM). This work [37] was only mentioned here to point out to all authors the need to present reliable data in their works, aiming always to show results with standard error less than 10%.

In other lines of work, several studies (e.g., Reddy et al. [83], Chimnoi et al. [98] and Endringer et al. [101]) assessed the antitumor potential of isolated compounds from *Hedychium* extracts without following the best guidelines for evaluating the cytotoxic potential of compounds. In fact, the authors did not test a reference compound in the same experimental conditions, and did not test the isolated compounds against a non-tumor cell line, which makes it difficult to draw conclusions. Regrettably, without these results, it is not possible to conclude about the efficacy and selectivity of the isolated compound compared to the drugs already available on the market.

**Figure 2:**
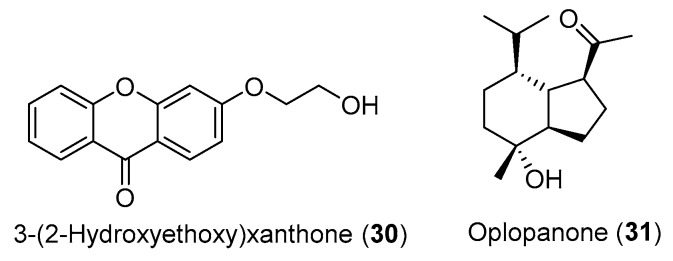
Chemical structure of the compounds **30** and **31**.

In addition to compounds **28** and **29** (Figure 1), Carvalho and colleagues [104] also isolated the compounds 3-(2-hydroxyethoxy)xanthone (**30**) and oplopanone (**31**) from *H. gardenerianum* rhizome acetone extract, but the two compounds (Figure 2) do not present any reported activity and, therefore, were not included in Table 2. Since they belong to families of organic compounds well-known for their broad spectrum of activities (flavonoids and terpenes) [107], it would be worth investigating the biological activity of these compounds.

It is a fact that the availability of a specific compound in a plant can depend on several factors, like the geographic location where the plant developed [108] and/or the season when it was harvested [109]. Thus, different studies can present different percentages of the total content of the same compound which makes it difficult sometimes to make comparisons between the same plants. This fact is particularly relevant with regard to essential oils, where the majority of published studies refers to quantitative chemical analysis. These studies reveal a complex composition and a huge variability in the content of each compound, depending on geographic, seasonal and species factors, which is reflected in the variability of the biological activity level of the respective essential oils, already highlighted in point 2.

**Figure 3:**
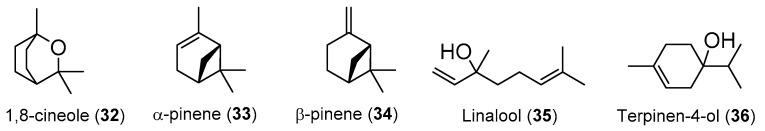
Chemical structure of the compounds referred on Table 3.

*Hedychium* species are not different, with several compounds being identified with distinct percentages on its essential oils. However, a deeper analysis of the published works allows to identify some compounds that, with some slight differences, appear repeatedly as the most abundant compounds in their essential oils. In Table 3 are gathered the five most abundant compounds identified in essential oils from *Hedychium* species as well as their activities and the species where they have already been identified. The respective structures are presented on Figure 3.

As it is possible to see on Table 3, linalool (**35**) proved to have promising antitumor potential since it presented cytotoxicity against U937 cell line (i.e., IC_50_ = 2.59 µM), better than the positive control 5-FU against the same cell line (i.e., IC_50_ = 4.86 µM). It would have been interesting if the authors had tested the compounds cytotoxicity against a non-tumor cell line, but unfortunately that was not the case.

From the five most abundant and most frequent present compounds in essential oils of *Hedychium* species, β-pinene (**34**) is the most widespread compound among species being identified in 12 *Hedychium* species, mainly rhizomes but also in some cases from flower and leaf essential oil. The compounds α-pinene (**33**) and linalool (**35**), exhibit a broad range of bioactivities, being anti-acetylcholinesterase, anti-allergic, antidepressive, antidiabetic, anti-inflammatory, antimicrobial, antitumor, fumigant and neuroprotective agents.

The antimicrobial activity of α-pinene (**33**), β-pinene (**34**) reported by Leite et al. [115] presented in Table 3 should be noted, which appears as µL/mL and the authors do not provide the necessary data to convert it to µM or µg/mL. Thus, it is impossible to compare the exhibited activity with other published results and even to compare with the positive control used in this study.

**Figure 4:**
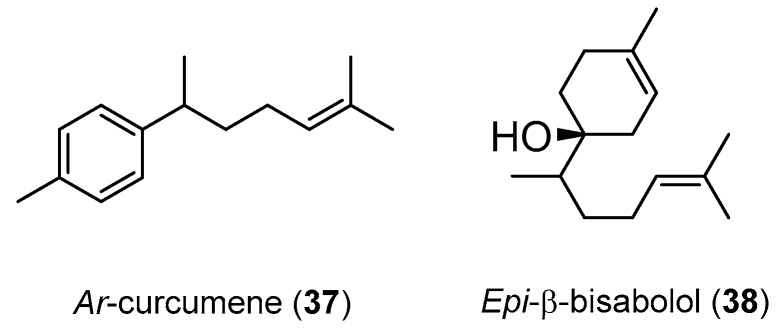
Chemical structure of the compounds **37** and **38**.

Despite not being so abundant as the compounds referred in Table 3, the isolation of two compounds from *H. larsenii* rhizomes essential oil could be mentioned, i.e., *ar*-curcumene (**37**) and *epi*-β-bisabolol (**38**) (Figure 4), that presented insecticide properties against diseases mosquito vectors larvae *A. stephensi*, *A. aegypti* and *C. quinquefasciatus* [94]. The results show that the most affected vector was *A. stephensi* with compounds **37** and **38** presenting a LC_50_ values of 51.65 and 66.02 µM, respectively. Unfortunately, the lack of a tested reference compound is a handicap in this work.

Taking together, Table 2 and Table 3 offer a summary view point of the works carried out in recent years that permitted the isolation of some compounds from *Hedychium* genus, being ascertained their bioactivities. This allows to easily identify where there is work already successfully developed and which paths have not yet been explored.

## 4. Conclusions

*Hedychium* genus is undoubtedly proven to be a valuable group of medicinal plants, being present in several folk medicines around the world where it is known to treat allergies, cancer, diabetes, inflammation, rheumatism and skin problems, as well as being also used as an analgesic, antimicrobial, anti-helminthic, antioxidant and insect repellent. In addition, some *Hedychium* species are part of human diet, being cooked as a vegetable, used as a spice or drunk as a beverage.

Several works explored *Hedychium* species in order to confirm if and how effectively these plants exert the reported biological effects on folk medicine, studying their essential oils, extracts and their isolated compounds. Taking into account the results of the literature in recent years, *Hedychium* species have been proven to possess interesting pharmaceutical activities, i.e., anti-acetylcholinesterase, antidiabetic, anti-inflammatory, antimicrobial, antioxidant, antitumor and hepatoprotective, as well as having potential to develop insecticides.

Phytochemical works have been carried out in *Hedychium*, mainly on *H. coronarium* and *H. spicatum*, but also on other less known species, leading to the isolation of interesting compounds that, in some cases, proved to be better than reference compounds. An example is coronarin D (**7**), possessing antifungal, antitumor and antibacterial properties, being more effective than the positive control oxacillin against *B. cereus* in antibacterial assays. Isocoronarin D (**11**), villosin (**26**) and linalool (**35**) can be pointed out as very promising antitumor compounds since they exhibited better cytotoxicity towards tumor cell lines than the reference compounds used, and in case of villosin (**26**) without toxicity on non-tumor cell line. Furthermore, the most bioactive compounds found in *Hedychium* essential oils can be highlighted as α-pinene (**33**) and linalool (**35**), since they are reported as presenting a wide spectrum of bioactivities. In addition, being identified in 12 different *Hedychium* species to this date, β-pinene (**34**) is the most widespread compound in *Hedychium* essential oils.

*Hedychium* species as proved to be a very rich genus that can still have a lot to offer to the scientific community. Moreover, the discovery in recent years of four new *Hedychium* species (i.e., *Hedychium chingmeianum* N.Odyuo and D.K.Roy [128], *Hedychium putaoense* Y.H.Tan and H.B.Ding [129], *Hedychium viridibracteatum* X.Hu [130] and *Hedychium ziroense* V.Gowda and Ashokan [131], may bring new compounds with pharmaceutical potential to the equation.

## Figures and Tables

**Table 1:** *Hedychium* species with reported traditional medicinal use.

*Hedychium* Species	Geographical Origin of the Reported Traditional Use	Traditional Medicinal Use	Preparation and/or Administration
*Hedychium* sp. [23]	Myanmar [23]	Cuts and wounds [23]	Cataplasms of crushed leaves and rhizomes [23]
		Weak blood circulation and to accelerate postpartum recovery [23]	Decoction of rhizomes is drunk [23]
*Hedychium coccineum* Buch.-Ham. ex Sm.	India [24]	Jaundice [24]	Decoction of rhizomes [24]
*H. coronarium*	Brazil [25,26]	Anti-inflammatory and sedative [25]	Leaves infusion [25,26]
		Headache and fever [26]	
	China [27]	Diabetes, headache, inflammation, rheumatism and skin diseases [27]	Rhizomes [27]
	Colombia [28]	Snake bites [28]	Decoction of rhizomes [28]
	India [16,29,30,31]	Stimulant tonic, carminative, headache, fever, diphtheria and diabetes [16,29,30]	Grinded rhizomes [16,29,30]
		Abdominal pain [31]	10 g of sun-dried rhizome powder mixed with cooked vegetables [31]
	Malaysia [32]	Indigestion and abdominal pain [32]	Boiled leaves with betel nut are eaten [32]
	Mauritius [33]	Carminative, cordial, emmenagogue, diuretic and toothache [33]	Decoction of rhizomes [33]
		Rubefacient [33]	Cataplasm of fresh rhizomes [33]
		Rheumatism [33]	Rub affected areas with paste from crushed rhizomes cooked in mustard oil with garlic and crushed camphor bark [33]
	Nicaragua [34]	Snake bites [34]	Decoction of rhizomes [34]
	Peru [35]	Soothing and rheumatism [35]	Bath is prepared with the aerial part [35]
	Thailand [16,36]	Sore and stiff joints [16]	Application of boiled leaves in affect areas [16]
		Tonsillitis [16]	Decoction of the stem is gargled [16]
		Mosquito repellent [36]	Oil from the plant [36]
	Vietnam [37]	Diabetes, headache, inflammation, rheumatism and skin diseases [37]	Rhizomes [37]
*Hedychium cylindricum* Ridl.	Malaysia [38]	Antirheumatic, febrifuge, tonic, treatment of skin diseases and wounds [38]	Rhizomes [38]
*Hedychium ellipticum* Buch.-Ham. ex Sm.	Nepal [39]	Fever [39]	Five teaspoons twice a day of rhizome juice [39]
*Hedychium flavescens* Carey ex Roscoe	Madagascar [40]	Caries [40]	Squeezed leaves liquid is applied in cotton and then placed in the affected cavity [40]
	Mauritius [33]	Rheumatism [33]	Rub affected areas with paste from crushed rhizomes cooked in mustard oil [33]
*Hedychium longicornutum* Griff. ex Baker	Malaysia [41]	Intestinal worms and earache [41]	Macerated roots or the whole plant [41]
*Hedychium spicatum* Sm.	India [21,42,43,44]	Bad breath, bronchitis, blood diseases, hiccough and vomiting [42]	3 to 4 g of rhizome powder two times a day [42]
		Asthma, body pain, inflammation and laxative [43]	1 g dried rhizome powder twice a day [43]
		Diarrhea, fever, liver problems and pain [21]	Spoonful of dried rhizome powder thrice a day [21]
		Expectorant, stimulant, stomachic, tonic and vasodilator [21]	Cup of the rhizome decoction twice a day [21]
		Snake bites [44]	
	Nepal [39]	Indigestion and high fever [39]	Decoction of rhizome three to five teaspoons twice a day [39]

**Table 2:** Secondary metabolites isolated from *Hedychium* extracts with proven activities.

Compound	Extract	*Hedychium* Source	Activity *
Hedyforrestin B (**1**)	Hexane [95]	*H. gardnerianum* rhizome [95]; *Hedychium longipetalum* X.Hu and N.Liu rhizome [96]	Antitumor against NCI-H187 cell line (IC_50_ = 3.10 µM; Vero cell line IC_50_ = 45.07 µM with SI of 14.5; Ellipticine IC_50_ = 1.79 µM) [95]; Anti-inflammatory by NO inhibition (IC_50_ = 20.60 µM **; MG132 ^#^ IC_50_ = 0.17 µM **) [96]
Hedyforrestin C (**2**)	Dichloromethane [95]; Methanol [37]	*H. gardnerianum* rhizome [95]; *H. coronarium* rhizome [37]; *H. longipetalum* rhizome [96]	Antitumor against NCI-H187 cell line (IC_50_ = 2.46 µM; Vero cell line IC_50_ = 11.88 µM with SI of 4.8; Ellipticine IC_50_ = 1.79 µM) [95]; Anti-inflammatory by NO inhibition (IC_50_ = 8.33 µM **; MG132 ^#^ IC_50_ = 0.17 µM **) [96]
Hedylongnoid A (**3**)	†	*H. longipetalum* rhizome [96]	Anti-inflammatory by NO inhibition (IC_50_ = 22.84 µM **; MG132 ^#^ IC_50_ = 0.17 µM **) [96]
Hedylongnoid B (**4**)	†	*H. longipetalum* rhizome [96]	Anti-inflammatory by NO inhibition (IC_50_ = 16.79 µM **; MG132 ^#^ IC_50_ = 0.17 µM **) [96]
Hedylongnoid C (**5**)	†	*H. longipetalum* rhizome [96]	Anti-inflammatory by NO inhibition (IC_50_ = 17.50 µM **; MG132 ^#^ IC_50_ = 0.17 µM **) [96]
Yunnancoronarin A (**6**)	Chloroform [83]; Hexane [95]	*H. gardnerianum* rhizome [95]; *H. spicatum* rhizome [83]; *H. longipetalum* rhizome [96]	Antitumor against NCI-H187 cell line (IC_50_ = 36.78 µM; Vero cell line IC_50_ = 108.61 µM with SI of 2.9; ellipticine IC_50_ = 1.79 µM) [95]; Antitumor against Colo-205 cell line (IC_50_ = 90.35 ± 0.10 µM **) [83]; Antitumor against CHO cell line (IC_50_ = 59.55 ± 3.93 µM **) [83]; Anti-inflammatory by NO inhibition (IC_50_ = 1.86 µM **; MG132 ^#^ IC_50_ = 0.17 µM **) [96]
Coronarin D (**7**)	Dichloromethane [97]; Ethanol [80]; Hexane [98]; Methanol [99]	*H. coronarium* rhizome [80,97,98,99]	Antitumor against S102 cell line (IC_50_ = 25.13 µM **) [98]; Antitumor against P388 cell line (IC_50_ = 4.40 µM **; Etoposide IC_50_ = 0.12 µM **) [97]; Antibacterial against *B. cereus* (MIC = 19.63 µM **; oxacillin MIC = 62.28 µM **) [100]; Antifungal against *Cryptococcus albidus* (MIC = 78.52 µM **; Amphotericin B MIC = 0.84 µM **) [100]
Coronarin D ethyl ether (**8**)	Hexane [98]	*H. coronarium* rhizome [98]	Antitumor against HepG2 cell line (IC_50_ = 46.18 µM **) [98]
Coronarin B (**9**)	Dichloromethane [97]; Hexane [98]	*H. coronarium* rhizome [97,98]	Antitumor against MOLT-3 cell line (IC_50_ = 1.32 µM **; Etoposide IC_50_ = 0.03 µM **) [97]
Coronarin D acetate (**10**)	Dichloromethane [97]	*H. coronarium* rhizome [97]	Antitumor against P388 cell line (IC_50_ = 4.72 µM **; etoposide IC_50_ = 0.12 µM **) [97]
Isocoronarin D (**11**)	Dichloromethane [97]; Ethanol [101]; Hexane [98]	*H. coronarium* rhizome [97,98,101]	Antitumor against P388 cell line (IC_50_ = 2.14 µM **; etoposide IC_50_ = 0.12 µM **) [97]; Antitumor against HepG2 cell line (IC_50_ = 54.7 ± 0.3 µM) [101]
Benzoyl eugenol (**12**)	Ethanol [101]	*H. coronarium* rhizome [101]	Antitumor against HEK293 by NF-ĸB inhibition (IC_50_ = 32.5 ± 4.9 µM) [101]
Ethoxycoronarin D (**13**)	Ethanol [101]	*H. coronarium* rhizome [101]	Cancer chemo preventive by COX-1 inhibition (IC_50_ = 3.8 ± 0.1 µM) [101] Antitumor against HEK293 by NF-ĸB inhibition (IC_50_ = 3.2 ± 0.3 µM) [101]
Methoxy-coronarin D (**14**)	Ethanol [101]	*H. coronarium* rhizome [101]	Cancer chemo preventive by COX-1 inhibition (IC_50_ = 0.9 ± 0.0 µM) [101] Antitumor against HEK293 by NF-κB inhibition (IC_50_ = 7.2 ± 0.3 µM) [101]
Hedychiumin (**15**)	Methanol [102]	*H. coronarium* aerial part [102]	Antitumor against P388D1 cell line (IC_50_ = 17.15 ± 1.92 µM **; doxorubicin IC_50_ = 0.74 ± 0.11 µM **) [102]
Calcaratarin A (**16**)	Methanol [102]	*H. coronarium* aerial part [102]	Antitumor against P388D1 cell line (IC_50_ = 24.56 ± 1.92 µM **; doxorubicin IC_50_ = 0.74 ± 0.11 µM **) [102]
Coronarin A (**17**)	Hexane [95]; Methanol [102]	*H. gardnerianum* rhizome [95]; *H. coronarium* aerial part [102]	Antitumor against NCI-H187 cell line (IC_50_ = 40.77 µM; Vero cell line IC_50_ = 150.45 µM with SI of 3.7; ellipticine IC_50_ = 1.79 µM) [95]; Antitumor against DLD-1 cell line (IC_50_= 41.61 ± 6.32 µM **; doxorubicin IC_50_ = 0.39 ± 0.07 µM **) [102]
9-Hydroxy hedychenone (**18**)	Chloroform [83]	*H. spicatum* rhizome [83]	Antitumor against Colo-205 cell line (IC_50_ = 76.40 ± 0.03 µM **) [83]; Antitumor against CHO cell line (IC_50_ = 49.87 ± 0.29 µM **) [83]
Hedychilactone B (**19**)	Chloroform [83]	*H. spicatum* rhizome [83]	Antitumor against Colo-205 cell line (IC_50_ = 86.55 ± 0.06 µM **) [83]; Antitumor against CHO cell line (IC_50_ = 60.94 ± 0.25 µM **) [83]
Hedychilactone C (**20**)	Chloroform [83]	*H. spicatum* rhizome [83]	Antitumor against Colo-205 cell line (IC_50_ = 111.73 ± 0.09 µM **) [83]; Antitumor against CHO cell line (IC_50_ = 70.82 ± 0.24 µM **) [83]
Hedychilactone D (**21**)	Chloroform [83]	*H. spicatum* rhizome [83]	Antitumor against Colo-205 cell line (IC_50_ = 36.41 ± 0.09 µM **) [83]; Antitumor against CHO cell line (IC_50_ = 23.27 ± 3.39 µM **) [83]
Chrysin (**22**)	Chloroform [83]	*H. spicatum* rhizome [83]	Antitumor against Colo-205 cell line (IC_50_ = 117.25 ± 0.24 µM **) [83]; Antitumor against CHO cell line (IC_50_ = 83.94 ± 4.37 µM **) [83]
Teptochrysin (**23**)	Chloroform [83]	*H. spicatum* rhizome [83]	Antitumor against Colo-205 cell line (IC_50_ = 122.63 ± 0.11 µM **) [83]; Antitumor against CHO cell line (IC_50_ = 110.86 ± 0.15 µM **) [83]
Hedychin C (**24**)	Ethanol [103]	*H. forrestii* rhizome [103]	Antitumor against XWLC-05 cell line (IC_50_ = 53.6 µM) [103]
Coronarin E (**25**)	Hexane [95]	*H. gardnerianum* rhizome [95]	Antitumor against NCI-H187 cell line (IC_50_ = 49.73 µM; Vero cell line IC_50_ = 164.19 µM with SI of 3.3; ellipticine IC_50_ = 1.79 µM) [95]
Villosin (**26**)	Dichloromethane [95]	*H. gardnerianum* rhizome [95]	Antitumor against NCI-H187 cell line (IC_50_ = 0.40 µM; Vero cell line IC_50_ > 166.42 μM with SI > 416; ellipticine IC_50_ = 1.79 µM) [95]
Yunnancoronarin B (**27**)	Hexane [95]	*H. gardnerianum* rhizome [95]	Antitumor against NCI-H187 cell line (IC_50_ = 44.57 µM; Vero cell line IC_50_ = 106.21 µM with SI of 2.4; ellipticine IC_50_ = 1.79 µM) [95]
1-Hydroxyxanthone (**28**)	Acetone [104]	*H. gardenerianum* rhizome [104]	Anti-depressant by MAO-A inhibition (IC_50_ = 0.31 ± 0.05 µM) [105]
Salicylic acid (**29**)	Acetone [104]	*H. gardenerianum* rhizome [104]	Anti-hemorrhagic (IC_50_ = 0.20 µM) [106]

* Only the highest activity value; ** Value after unit conversion from µg/mL to µM; ^#^ MG132-carbobenzoxy-Leu-Leu-leucinal positive control; ^†^ The authors do not indicate the extract prepared.

**Table 3:** The five most frequent and abundant chemical compounds identified in essential oils from *Hedychium* species.

Compound	Activity *	*Hedychium* Source
1,8-Cineole (**32**)	Antifungal against *C. albicans* (MIC = 203 µM **; nystatin MIC = 135 µM **) [110]; Insecticide against *Rhodnius prolixus* (KT_50_ = 117.2 min for 100 µL dose) [111]	*H. coronarium* rhizome [16,112]; *Hedychium flavescens* Carey ex Rosc. rhizome [112]; *Hedychium flavum* Roxb rhizome [112]; *H. gardnerianum* rhizome [74]; *H. gracile* rhizome [73,112]; *H. greenii* rhizome [73]; *H. larsenii* rhizome [94]; *H. spicatum* rhizome [67,109,112]
α-Pinene (**33**)	Anti-acetylcholinesterase (IC_50_ = 10.50 ± 0.51 µM **; ursolic acid IC_50_ = 0.416 ± 0.003 µM **) [50]; Anti-allergic (dose of 10 mg/kg on mouse) [113]; Antidiabetic (dose of 0.25 mL/kg on mouse) [114]; Anti-inflammatory (mouse ED_50_ = 0.039 mL/kg) [114]; Antimicrobial against *Streptococcus pneumoniae* (MIC = 5 µL/mL; gentamicin MIC = 21 µM **) [115]; Antitumor against A549 cell line (IC_50_ = 161.56 ± 12.85 µM ** [116]	*H. coccineum* (syn. *Hedychium aurantiacum* Roscoe) rhizome [112]; *H. coronarium* flower [117] and rhizome [16,112]; *H. flavescens* rhizome [112]; *H. flavum* rhizome [112]; *H. gardnerianum* flower [118], leaf [118] and rhizome [112]; *H. greenii* rhizome [73,112]; *H. matthewii* rhizome [65]; *H. spicatum* rhizome [109]
β-Pinene (**34**)	Antimicrobial against *S. pneumoniae* (MIC = 20 µL/mL; gentamicin MIC = 21 µM **) [115]; Antitumor against HCT-8 cell line (IC_50_ = 176.9 ± 2.9 µM **) [119]	*H. coccineum* (syn. *H. aurantiacum*) rhizome [112]; *H. coronarium* flower [117] and rhizome [16,108,112]; *H. ellipticum* rhizome [112]; *H. flavescens* rhizome [112]; *H. flavum* rhizome [112]; *H. gardnerianum* flower [118], leaf [118] and rhizome [112]; *H. gracile* rhizome [73,112]; *H. greenii* rhizome [73]; *H. larsenii* rhizome [94]; *H. matthewii* rhizome [65]; *H. spicatum* rhizome [67,109]; *Hedychium thyrisiforme* Smith. rhizome [112]
Linalool (**35**)	Antibacterial against *Bacillus mycoides* (10 µL cause 11 mm inhibition zone; 10 µL of penicillin = 12 mm inhibition zone) [120] Antidepressive (dose of 100 mg/kg on mouse) [121]; Anti-inflammatory (dose of 30 mg/kg on mouse) [122]; Antitumor against U937 cell line (IC_50_ = 2.59 µM; 5-FU IC_50_ = 4.86 µM) [123]; Fumigant against *Tribolium confusum* larvae (LC_50_ = 14.198 μL/L of air) [124]; Neuroprotective (100 μM reduced 30% OHSC cell death) [125]	*H. coronarium* flower [117] and rhizome [108]; *H. flavum* rhizome [112]; *H. larsenii* rhizome [94]; *H. matthewii* rhizome [65]; *H. spicatum* rhizome [67,109]
Terpinen-4-ol (**36**)	Antibacterial against *Burkholderia pyriocinia* (10 µL cause 8 mm inhibition zone; 10 µL of penicillin cause 9 mm inhibition zone) [120]; Antifungal against *Histoplasma capsulatum* (MIC = 129.70 µM **; AMB MIC = 0.54 µM **) [126]; Antitumor against MCF-7 cell line (IC_50_= 18.02 µM **; doxorubicin IC_50_ = 1.29 µM **) [127]	*H. ellipticum* rhizome [112]; *H. gracile* rhizome [73,112]; *H. larsenii* rhizome [94]; *H. matthewii* rhizome [65]; *H. thyrisiforme* rhizome [112]

* only the highest activity value; ** Value after unit conversion from µg/mL to µM.

## Abbreviations

A-431	Human epidermoid carcinoma
A549	Human lung adenocarcinoma
ABTS	2,2′-azino-bis(3-ethylbenzothiazoline-6-sulphonic acid)
AChE	Acetylcholinesterase
AgNPs	*H. coronarium*-synthesized silver nanoparticles
BHT	2,6-di-tert-butyl-4-methylphenol
BMDC	Bone marrow-derived dendritic cells
C57BKS^db/db^	Strain of laboratory mouse with a mutation that results in chronic hyperglycemia, pancreatic beta cell atrophy, low insulin level and obesity
CAT	Catalase
CHO	Chinese hamster ovary cells
Colo-205	Colorectal adenocarcinoma
COX-1	Cyclooxygenase 1
DLD-1	Human colorectal carcinoma
DPPH	1,1-Diphenyl-2-picrylhydrazyl
EC_50_	Half maximal effective concentration
ED_50_	Half maximal effective dose
GPx	Glutathione peroxidase
HeLa	Human cervical carcinoma
HepG2	Human hepatocellular carcinoma
HL-60	Human acute promyelocytic leukemia
HuCCA-1	Human cholangiocarcinoma
HUVEC	Human umbilical vascular endothelial cells
IC_50_	Half maximal inhibitory concentration
IFN-γ	Interferon gamma
IL-6	Interleukin 6
Il-12p40	Interleukin-12 subunit p40
KB	Human epidermoid carcinoma
KT_50_	Knockdown time 50%
LC_50_	Lethal concentration that kills 50% of exposed organisms
LNCaP	Human prostate adenocarcinoma
LPS	Lipopolysaccharide
MAO-A	Monoamine oxidase A
MCF-7	Human breast adenocarcinoma
MDA-MB-231	Human breast adenocarcinoma
MG132	Carbobenzoxy-Leu-Leu-leucinal
MIC	Minimum inhibitory concentration
MOLT-3	Human acute lymphoblastic leukemia T-lymphoblasts
MTT	3-(4,5-Dimethylthiazol-2-yl)-2,5-diphenyltetrazolium bromide
NCI-H187	Human classic small cell lung carcinoma
NF-κB	Nuclear factor kappa-B
NO	Nitric oxide
OHSC	Organotypic hippocampal slice cultures
P388	Mouse lymphoma neoplasm
P388D1	Murine macrophage-like lymphoma
RAW 264.7	Murine macrophage
ROS	Reactive oxygen species
S102	Human hepatocellular carcinoma
SI	Selectivity index
SOD	Superoxide dismutase
STZ	Streptozotocin
T2DM	Type 2 *diabetes mellitus*
T-47D	Human hormone-dependent breast cancer
TNF-α	Tumor necrosis factor α
U937	Human histiocytic lymphoma
Vero	African green monkey kidney epithelial cells
XWLC-05	Human lung adenocarcinoma
